# Edaravone Alleviates Traumatic Brain Injury by Inhibition of Ferroptosis via FSP1 Pathway

**DOI:** 10.1007/s12035-024-04216-2

**Published:** 2024-05-11

**Authors:** Haoyu Shi, Libiao Song, Yonghui Wu, Ruonan Shen, Chenxu Zhang, Xingzhi Liao, Qiuhong Wang, Jie Zhu

**Affiliations:** 1 Department of Neurosurgery, Wuxi Clinical College of Anhui Medical University (The 904th Hospital of PLA)/Fifth Clinical Medical College of Anhui Medical University, Wuxi, 214044 Jiangsu Province China; 2 Department of Anaesthesiology, Wuxi Clinical College of Anhui Medical University (The 904th Hospital of PLA)/Fifth Clinical Medical College of Anhui Medical University, Wuxi, 214044 Jiangsu Province China; 3 Department of Ophthalmology, Wuxi Second Hospital Affiliated to Jiangnan University, Wuxi, 214002 Jiangsu Province China; 4 Department of Neurosurgery, The Second People’s Hospital of Lu’an, Lu’an, 237000 Anhui Province China

**Keywords:** Traumatic brain injury, Ferroptosis, Mechanical injury, Edaravone, Ferroptosis suppressor protein 1

## Abstract

Traumatic brain injury (TBI) is a highly severe form of trauma with complex series of reactions in brain tissue which ultimately results in neuronal damage. Previous studies proved that neuronal ferroptosis, which was induced by intracranial haemorrhage and other reasons, was one of the most primary causes of neuronal damage following TBI. However, the association between neuronal mechanical injury and ferroptosis in TBI and relevant treatments remain unclear. In the present study, we first demonstrated the occurrence of neuronal ferroptosis in the early stage of TBI and preliminarily elucidated that edaravone (EDA), a cerebroprotective agent that eliminates oxygen radicals, was able to inhibit ferroptosis induced by TBI. A cell scratching model was established in PC12 cells, and it was confirmed that mechanical injury induced ferroptosis in neurons at the early stage of TBI. Ferroptosis suppressor protein 1 (FSP1) plays a significant role in inhibiting ferroptosis, and we found that iFSP, a ferroptosis agonist which is capable to inhibit FSP1 pathway, attenuated the anti-ferroptosis effect of EDA. In conclusion, our results suggested that EDA inhibited neuronal ferroptosis induced by mechanical injury in the early phase of TBI by activating FSP1 pathway, which could provide evidence for future research on prevention and treatment of TBI.

## Introduction

In recent years, traumatic brain injury (TBI) has emerged as a primary cause of disability or mortality in children and adolescents. Approximately 69 million people worldwide suffer from TBI every year [1]. In China, TBI is a notable critical ailment, ranking second only to limb injuries, with the main causative factors of traffic accidents, falls, stumbles, and firearms. Although clinical diagnosis and treatment of TBI have made progress, TBI still has the highest mortality and disability rates among all body injuries [2, 3]. Due to its significant impact on mortality and disability, TBI has become a key area in neurosurgical research. As deeper research on the mechanisms of TBI, it has become evident that the neuronal cytological irreversible damage is a crucial factor for the unfavourable prognosis of TBI [4]. Therefore, preventing and alleviating neuronal injury after trauma has become a critical way for improving the prognosis of TBI. Neurons undergo a series of reactions, such as the release of excitatory neurotransmitters, calcium overload, free radical destruction, oxidative stress, activation of apoptotic genes, and an inflammatory response in the early stages of TBI, leading to severe damage [5, 6]. However, brain protective agents aimed at above targets have not achieved the expectant effects in clinical trials.

Ferroptosis is a novel form of programmed cell death, firstly mentioned from Dixon’s team in 2012, which has unique morphological and biochemical features distinguished from apoptosis, pyroptosis, and autophagy [7]. A significant feature of cellular ferroptosis is the modification of mitochondrial morphology, the location of which reactive oxygen species (ROS) generate [7]. Ferroptosis is dependent on the release of superoxide anion (O2• −) from dysfunctional mitochondrial and the Fe^2+^ accumulation from disordered iron metabolism, which induces Fenton and Haber–Weiss reactions. These reactions lead to excessive production of more cytotoxic hydroxyl radicals (OH•) and affect a series of cascade reactions of DNA replication, transcription, and translation. Then, lipid peroxidation of unsaturated fatty acids (PUFA) is catalysed, and cell death is induced due to cell membrane lysis [8]. Meanwhile, initiation of ferroptosis is accompanied by impairment of antioxidant systems, including reduction of GPX4 which is a critical enzyme in the regulation of glutathione (GSH) system, the inhibition of the XC-system which relies on the synthesis of GSH, and the decline in the expression of ferroptosis suppressor protein 1 (FSP1).

Recently, it has been reported that TBI strongly correlates with ferroptosis [9, 10]. A retrospective analysis of TBI patients exhibited that disordered internal iron metabolism environment caused by iron-regulated protein dysfunction was associated with Glasgow Coma Scale (GCS) and intensive care unit (ICU) mortality in TBI patients [11]. Imaging perspective of TBI patients revealed an increase iron content in brain post TBI [12]. It is noteworthy that the brain contains the highest levels of PUFAs, providing adequate material for lipid peroxidation, strongly enhancing neurons susceptible to ferroptosis [13]. Based on the potential role of ferroptosis, new targeted therapeutic treatments were proposed to improve prognosis of TBI, including inhibition of neuronal sensitivity to ferroptosis. Thus, it is necessary to elucidate precise molecular mechanisms behind ferroptosis to exploit these treatments.

Mechanical neuronal injury, as the earliest and most direct mechanism of injury in TBI, serves as the primary cause of neuronal injury in TBI. It is commonly known that secondary neuronal injury is closely correlated with neuronal ferroptosis following TBI. Previous research has indicated that TBI leads to accumulation of red blood cells because of intracranial haemorrhage, then dissociative haemoglobin releases quantities of free iron under the effect of haemoglobin oxygenase. This internal environment iron ions provide fundamental material for iron-dependent Fenton reaction and Haber–Weiss reaction, producing toxic ROS, consequently resulting in ferroptosis in neurons [14–17]. However, whether mechanical neuronal injury, the earliest and most direct injury in TBI, correlated with neuronal ferroptosis in early TBI remains unclear.

The cerebroprotective agent edaravone (EDA) achieves brain protection by exerting its oxygen radical scavenging function and antagonising the occurrence of lipid peroxidation in neurons [18]. It is widely used in the treatment of stroke to improve neurological symptoms as well as dysfunction caused by cerebral infarction. In recent years, studies have demonstrated the efficacy of edaravone in the treatment of depression [19], vascular dementia [20], amyotrophic lateral sclerosis [21], and a range of central nervous system (CNS) disorders. However, there are no studies on the efficacy and mechanism of EDA therapy after TBI.

Ferroptosis suppressor protein 1 (FSP1), which was also known as apoptosis-inducing factor mitochondria-associated protein 2 (AIFM2), is an oxidase of NAD(P)H [22]. In 2019, Doll et al. found an additional pathway involving FSP1 that regulated ferroptosis independent of the traditional GSH pathway [23]. By performing NAD(P)H oxidase activity, it dehydrogenates NAD(P)H and delivers it to ubiquinone (CoQ_10_) in the cell membrane, which converts to its reduced form ubiquinol (CoQ_10_H_2_). The reduced CoQ_10_ inhibits peroxidation and reduces cellular susceptibility to ferroptosis. Although FSP1 has been suggested to be the second key pathway in the regulation of ferroptosis, the potential protective role of FSP1 in TBI has not been elucidated.

In this study, based on PC12 cell line scratch model and mice model that simulate mechanical injury of neurons, we explored the mechanism of neuronal ferroptosis after TBI in vitro and in vivo and evaluated the EDA therapeutic effect on inhibition of neuronal ferroptosis and prognosis improvement. Meanwhile, previous studies demonstrated that EDA could inhibit ROS generation by activating the Sirt1/Nrf2 pathway and thereby inhibiting ROS generation [19]. The association between Nrf2 and FSP1 has also been verified by the identification of two antioxidant response elements (AREs) upstream of the FSP1 transcriptional start site [24]. Given our previous studies on the protective role of FSP1 in neurological disorders [25], we validated whether EDA exert early brain protection by inhibiting ferroptosis after TBI through activating FSP1 pathway.

## Methods

### Mouse TBI Model

**Fig. 1:**
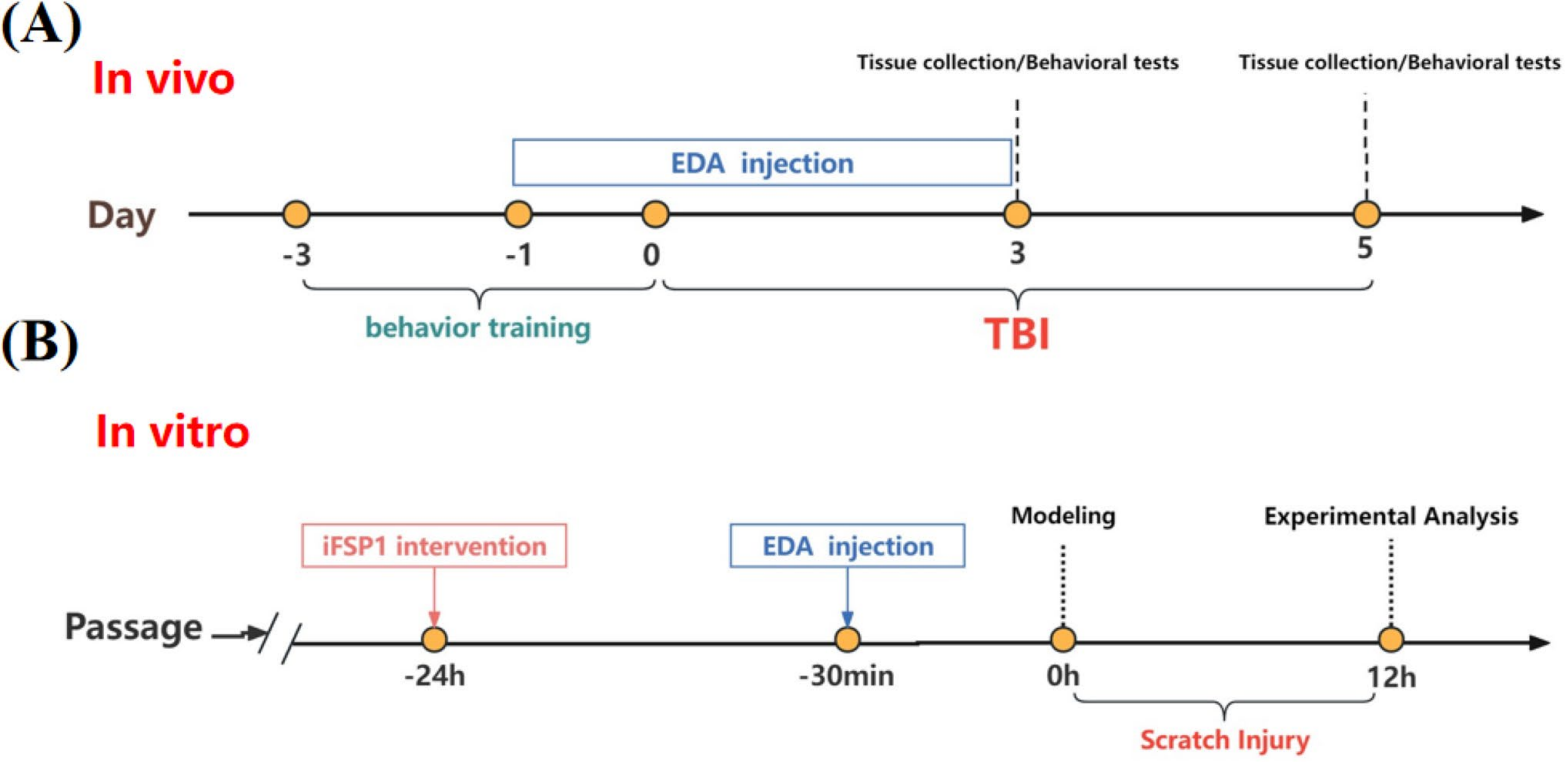
Schematic diagram of the experimental process

Eight-week-old female C57BL/6 J mice weighing 20–25 g were selected. Behavioural training was given to all experimental mice 3 days prior to controlled cortical impact instrument (CCI), which the methods were referred to previous experiments [26–28]. Randomly selected mice were anaesthetised with pentobarbital sodium (50 mg/kg, intraperitoneal injection), placed on a stereotaxic frame and secured with ear bars. Subsequently, a longitudinal skin incision was made to expose the skull, 2 mm to the left of the sagittal suture and 2 mm posterior to the fontanelle were selected as the site of striking, the skull covering the site of striking was abraded to create a 3 mm × 3 mm window of bone, and the bone fragments were removed taking care not to damage the endocranium. We selected appropriate parameters to establish the TBI model based on a previous study [26] and used a CCI for modelling, and the bone window was closed after the blow with the blow parameters (velocity, 4.0 ± 0.2 m/s; duration, 150 ms; depth, 1.5 mm), and then the scalp incision was sutured. The sham-operated group was sutured after opening the bone window. Mice were placed on a thermostatic blanket for observation for 1 h. Mice received edaravone (EDA) treatment (intraperitoneal injection of EDA 10 mg/kg) 24 h, 30 min before modelling, 2 h, 12 h, 24 h, 48 h, and 72 h after striking. EDA was purchased from Sigma-Aldrich (St. Louis, USA) and dissolved in 0.9% NaCI. Equal amounts of saline were given to the control and model groups. The dose of EDA intervention was determined with reference to previous studies [19, 29, 30]. We performed separate experiments for biochemical, histological, and behavioural assays. The number of animals used in each experiment is shown in the legend (Fig. 1A).

### In Vitro TBI

We chose the mechanical scratch model to simulate the mechanical injury of TBI and establish an in vitro TBI model. Many experiments have been applied for this model [31–33]. In this experiment, PC12 (highly differentiated) cell line (CL-0481, Pricella, Wuhan, China) was selected, which has nerve growth factor (NGF) receptor and produces a neuronal phenotype when induced by NGF (50 µg/ml) [34].

PC12 cells were cultured conventionally. The growth status and speed of the cells were observed every day, and the model was established when the cells grew normally to 60 ~ 70%. Methods were selected according to previous studies that scratches were made using the plastic tip of a 200-µL pipette with a scratch width of 1 mm, and two scratches were made horizontally and vertically in the petri dish. Prior to mechanical scratching of PC12 cells, the cell lines were intervened for 24 h with or without iFSP1, which was purchased from MedChemExpress (HY-136057, MCE), and then with or without edaravone (EDA) added 30 min before scratching. The cell growth status, the degree of healing of the scratched area, and other relevant assays were observed at 12 h after scratching (Fig. 1B).

### Western Blot Analysis

Mice were anaesthetised using pentobarbital sodium (70 mg/kg intraperitoneally), then the chest cavity was opened to expose the heart, the right ventricle was clipped, and PBS puffer was instilled via the left auricle. Brain tissue was taken, the striking side cortex was dissected out, and the injured cortex on the striking side (centre, ipsilateral cortical impact site; extent, 2 × 2 × 2 mm^3^) was collected and lysed by thorough shaking in lysis buffer containing PMSF (P105539, Aladdin, Shanghai, China) as well as RIPA (P0013B, Beyotime Biotechnology, Shanghai, China). As above, 120 ml of lysis buffer containing PMSF was added to each well of the cellular experimental specimen and placed on ice for 30 min for lysis with sufficient shaking. The samples were centrifuged with 12,000 r at 4 °C for 5 min, the supernatant was taken, and the samples were proportionally spiked according to the instructions of the BCA protein concentration determination kit (P0010, Beyotime Biotechnology, Shanghai, China), and the absorbance of OD562 was determined by a microplate reader (mμlISKANMK3, Thermo, USA). The absorbance of OD562 was measured by an enzyme marker (mμlISKANMK3, Thermo, USA), and the relevant linear regression equation was calculated.

Different molecular weight proteins were separated by performing electrophoresis on 10% or 15% SDS-PAGE gels. The proteins were then electrotransferred to PVDF membranes (IPVH00010, Millipore, USA), and the PVDF membranes were soaked with TBST containing 5% skimmed milk powder for room temperature shaker closure for 2 h. The membranes were then incubated with primary antibodies including FSP1 (BS-3759R, Bioss, Beijing, China), GPX4 (A13309, Abclonal, Wuhan, China), ACSL4 (A16848, Abclonal, Wuhan, China), Tfr1 (A5865, Abclonal, Wuhan, China), FTH (DF6278, Affinity, USA), COX2 (A1253, Abclonal, Wuhan, China), GAPDH (AB-P-R001, GOODHERE Biotech, Hangzhou, China) at 4 °C overnight. The PVDF membrane was then washed sufficiently using TBST for 5 times for 5 min each. The membrane was then incubated with secondary antibody on a shaker at room temperature for 2 h. The PVDF membrane was washed again with TBST. Target proteins were detected by chemiluminescent signals using ECL reagent (P1050, APPLYGEN, Beijing, China). Band densities were quantified by protein blotting using ImageJ analysis software and data were analysed normalised to GAPDH.

### TUNEL Staining

Mice were anaesthetised, the right ventricle was clipped, and a needle was inserted in the left auricle. Mice were perfused with PBS buffer, followed by perfusion fixation of tissues using 4% paraformaldehyde, and brain tissues were dissected out, put into 4% paraformaldehyde for a further 24 h and embedded in paraffin wax, and coronal Sects. (5 µm in thickness) were made at the site of the blow. For cellular specimens, slides crawled with cells were immersed in 4% paraformaldehyde solution and fixed at room temperature for 15 min, followed by washing with PBS three times. TUNEL positivity was detected using the TUNEL Apoptosis Detection Kit (E-CK-A322, Elabscience, Wuhan, China) according to the manufacturer’s instructions. Briefly, the permeabilisation of specimen cell membranes was increased using 0.2% Triton X-100 (PBS prepared) for 20 min at room temperature. The deparaffinised tissue sections were co-incubated with a working solution containing terminal deoxynucleotidyl transferase (TdT) enzyme and dUTP marker for 60 min at 37 °C protected from light, then the nuclei were re-stained with diluted DAPI, and finally, the sections were blocked, and the images were captured using a Nikon Fi3 biomicroscope and quantitatively analysed using the ImageJ software for quantitative analysis.

### Fluoro-Jade B (FJB) Staining

Paraffin sections of whole brain were taken by perfusion as previously described, and FJB staining was performed according to the manufacturer’s instructions (AG310, Millipore, Germany); in brief, the coronal sections were first immersed in an alkaline alcoholic solution for 5 min, followed by rinsing them in 70% ethanol for 2 min, and then the slides were washed in distilled water before being immersed in 0.06% potassium permanganate solution. The slides were washed with distilled water and incubated in the configured Fluoro-Jade B staining solution for 30 min, and the nuclei were restained using diluted DAPI. Images were captured using the Nikon Fi3 biomicroscope and quantitatively analysed using ImageJ software.

### Nissl Staining

For Nissl staining, we observed Nissl vesicles (essentially rough endoplasmic reticulum and ribosomes) in neuronal cells to determine neuronal function. Proceeding according to the manufacturer’s instructions (Beyotime Biotechnology, Shanghai, China), sections were washed and incubated in Nissl staining solution for 10 min at 50 °C. Images were captured using a Nikon Fi3 biomicroscope and quantitatively analysed using ImageJ software.

### Perls’ Blue Staining

Based on previous studies, we used Perls’ Prussian blue staining to target intracellular iron accumulation in animal experiments [35]. Briefly, after washing the brain sections three times with PBST, we covered the brain sections with a PBS solution of 0.3% Triton X-100 and incubated them for 5 min at room temperature. Then, the sections were incubated in the configured Perls’ solution (5% potassium ferrocyanide [Yeasen Biotech]/5% hydrochloric acid) for 30 min, after which they were again washed 3 times with PBST. Next, peroxidase activity was blocked using a methanol solution of 0.3% hydrogen peroxide, incubated for 15 min, and then washed again with PBST 3 times. Finally, the signal was generated by incubation in 3,3-diaminobenzidine (DAB) for 15 min and was restained using haematoxylin.

### Assay of Lipid Peroxide

In order to determine the level of lipid peroxidation to assess ferroptosis after TBI, we performed malondialdehyde (MDA), 4-HNE content, and ROS activity assays on the cortex around the contused area in the injury group and the corresponding area in the sham-operated group using the relevant kits (S0033, Beyotime Biotechnology, Shanghai, China) (S0131S, Beyotime Biotechnology, Shanghai, China) (LE-M1908, Lai Er Bio-Tech, Hefei, China), processed the samples according to the instructions, and used a fluorescent enzyme marker to measure the absorbance at 532 nm (ROS) and 450 nm (MDA,4-HNE), respectively.

### Behavioural Testing

Behavioural training was given to all experimental mice 3 days prior to CCI. This ensured that each mouse’s mNSS score was within the range of 0 to 1 point. Modified Neurological Severity Score (mNSS) was determined after 72 h in the sham or surgical group and in the treatment group. This neurological deficit score assesses primarily abnormal motor as well as sensory and reflex deficits and is scored on an 18-point neurological deficit scale. Its maximum score is 18, with higher scores indicating more severe neurological deficits.

### Transmission Electron Microscopy

Briefly, after treatment of the cells with trypsin, the cells were resuspended with glutaraldehyde and 0.1 mM PBS buffer, and then fixed at room temperature using 1% osmium tetroxide (Ted Pella Inc., USA) configured in 0.1 mM PBS buffer for 2 h. Subsequently, the specimens were dehydrated sequentially from high to low concentrations of alcohol and acetone. Finally, embedding polymerisation was performed, and sections were cut using an ultrathin slicer (Leica UC7, Leica, Germany). Observation of mitochondrial morphology was performed on transmission electron microscopy (HT7800, Hitachi, Japan).

### Immunofluorescence

The slides were washed with PBS, fixed with 4% paraformaldehyde for 15 min, and permeabilised with 0.5% TritonX-100 at room temperature. The slides were incubated overnight with primary antibody (BS-3759R, Bioss, China) and then with secondary antibody (BA1032, Boster, Wuhan, China) for 2 h. Finally, the nuclei were restained with DAPI, and the slides were blocked with a blocking solution resistant to fluorescence quencher. Observe and capture images under the Nikon Fi3 biomicroscope.

### Cell Viability Assay

PC12 cells in logarithmic growth phase with good growth status were inoculated into 6-well plates at 5 × 10^5 ^cells per well and cultured in adherence for 24 h, then the cells were subjected to relevant treatments (cell scratching, drug intervention). According to the manufacturer’s instructions, 10 µl cck8 (HYCCK8-500 T, HYCEZMBIO, Wuhan, China) was added to each well and incubated at 37 °C for 4 h; cell viability was evaluated by measuring the absorbance value OD450 in each well by microplate reader.

### Determination of Intracellular Iron Levels

To determine intracellular iron levels using the Iron Colorimetric Assay Kit (BC5415, Solarbio, Beijing, China), first, we centrifuged the sample and extracted the supernatant for testing. Then, we made a blank control group, a standard group, and an assay group according to the instructions of the kit. Two hundred microliter of test sample was added to the assay group, 200 µL of standard solution was added to the standard group, and 200 µL of mixture A was added to the blank group. One hundred microliter of the working solution was added and incubated at room temperature for 30 min, and finally, the absorbance was measured at 593 nm, and each experiment was repeated 6 times.

### Real-time Quantitative PCR

FSP1 mRNA levels were determined using real-time quantitative PCR. Briefly, total RNA was isolated using Trizol reagent (15596–026, Ambion, Beijing, China) according to the manufacturer’s protocol, and the concentration and purity of RNA were calculated using a microspectrophotometer to determine OD260, OD280, and the total RNA was reversed using HiScript® II Q RT SuperMix for qPCR (R233-01, VAZYME, Nanjing, China) to cDNA. Real-time PCR quantification was performed on all samples using SYBR Green mixture (Q111-02, Vazyme, Nanjing, China) to normalise the FSP1 mRNA level to the GAPDH mRNA expression of the internal control, and the relative quantification of the gene expression level was determined using the ΔΔCt method, with the primer list as follows.

**Table:** 

Gene	Primer	Sequence (5′-3′)	PCR products
Rat GAPDH	Forward	ACAGCAACAGGGTGGTGGAC	252 bp
	Reverse	TTTGAGGGTGCAGCGAACTT	
Rat FSP1	Forward	CCAGGGCAAAGTGATTGGCATAG	143 bp
	Reverse	GTTGGCAGGACACCTCGTTAA	

### Cell Flow-through ROS Level Assay

ROS levels in different subgroups of PC12 cells were tested using the H2DCF-DA probe (S0033M, Beyotime Biotechnology, Shanghai, China) according to the manufacturer’s instructions. The principle is due to the fact that DCFH-DA itself is non-fluorescent and freely crosses the cell membrane. It is hydrolysed intracellularly to DCFH, which is a good intracellular probe because it does not cross the cell membrane. DCFH is oxidised by intracellular ROS to form DCF with high fluorescence intensity, which reacts to the intracellular ROS level. The intensity of fluorescence before and after stimulation was detected in real time or time-point by time-point using 488 nm excitation wavelength and 525 nm emission wavelength. The relative fluorescence intensity was also used to statistically analyse the ROS levels between groups.

### Statistical Analysis

We statistically analysed the experimental data using a statistical analysis program called GraphPad Prism 8.0.2, which integrates and decomposes the data according to subgroups and provides means and standard deviations (mean ± SD) for correlation analyses, and assessed the data for normality and homogeneity of variances prior to the analysis by the Shapiro–Wilk test of normality and the Brown-Forsythe normality and homogeneity of variance. We used *t*-tests for comparisons between two groups and one-way ANOVA for comparisons between multiple groups. The threshold for statistical significance was set at *p* < 0.05.

#### Results

### Ferroptosis Occurred in Neurons at Early Stage of TBI

**Fig. 2:**
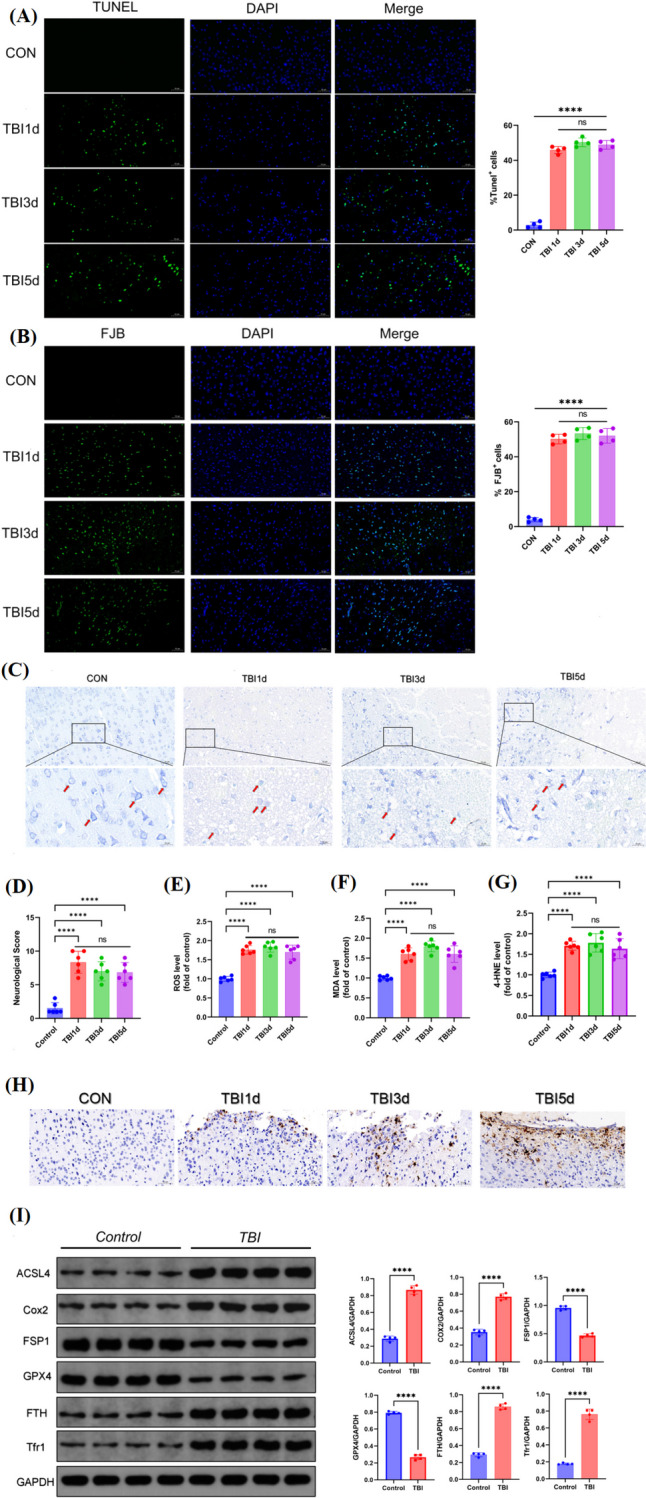
TBI-induced ferroptosis at early stage. **A** TUNEL staining (*n* = 4, scale bar 50 µm), **B** FJB staining (*n* = 4, scale bar 50 µm), and **C** Nissl staining of ipsilateral cortex of mice at 1 day, 3 day, and 5 day post TBI (scale bar 20 µm). **D** mNSS scores reflecting behavioural changes of mice at 1 day, 3 day, and 5 day post TBI (*n* = 6). **E** Lipid peroxidation indexes ROS (*n* = 6), **F** MDA, and **G** 4-HNE of mice brain after TBI (*n* = 6). **H** Iron accumulation in brain of mice after TBI (scale bar 50 µm). **I** Ferroptosis-associated protein detection of brain of mice 3 days post TBI (*n* = 4). *, **, ***, and **** indicate *p* < 0.05, 0.01, 0.001, and 0.0001, respectively

There is increasing evidence that irreversible neuronal damage is the main factor of poor prognosis of TBI [36]. Thus, we observed neuronal damage in the ipsilateral cortex of TBI mice by TUNEL staining, FJB staining, and Nissl staining at 1 day, 3 days, and 5 days post-strike. Results showed that a series of pathological changes like cytoplasmic crumpling or nuclear consolidation occurred in neurons, suggesting apoptosis; degenerative necrosis were induced (Fig. 2A and B). The protein synthesis function of neurons was reduced because of decreased number of Nissl bodies (Fig. 2C). Then, we also found significant increases of mNSS scores reflecting behavioural changes of mice from 1st day after TBI (Fig. 2D). Notably, neither neuronal pathology damage nor behavioural score was significantly changed with time.

In order to determine the presence of neuronal ferroptosis in the early stage of TBI, the lipid peroxidation indexes ROS, malondialdehyde (MDA), and 4-HNE were analysed. The results revealed that ROS, MDA, and 4-HNE raised significantly from the 1st day (Fig. 2E–G). Perls blue staining indicates iron accumulation, excessive of which could provide material for the ferroptosis-associated Fenton and Haber–Weiss reaction. We observed gradual accumulation of iron in brain tissues 1, 3, and 5 days post injury (Fig. 2H). Since iron metabolism disorder and lipid peroxidation are two major biochemical features of ferroptosis, we chose the 3rd day after TBI to detect the levels of ferroptosis-associated proteins or biomarkers: ACSL4, Cox2, Tfr1, FTH, FSP1, and GPX4 iron death. We found that the expression of ACSL4, Cox2, Tfr1, and FTH were significantly increased, while the expression of FSP1 and GPX4 was significantly decreased (Fig. 2I), suggesting occurrence of ferroptosis. Taken together, these data clearly demonstrated that irreversible damage occurred in neurons at early stage of TBI in mice and did not diminish with time, in which ferroptosis was one feature of neural injury.

### Edaravone Reduced Neuronal Damage and Inhibited Ferroptosis After TBI

**Fig. 3:**
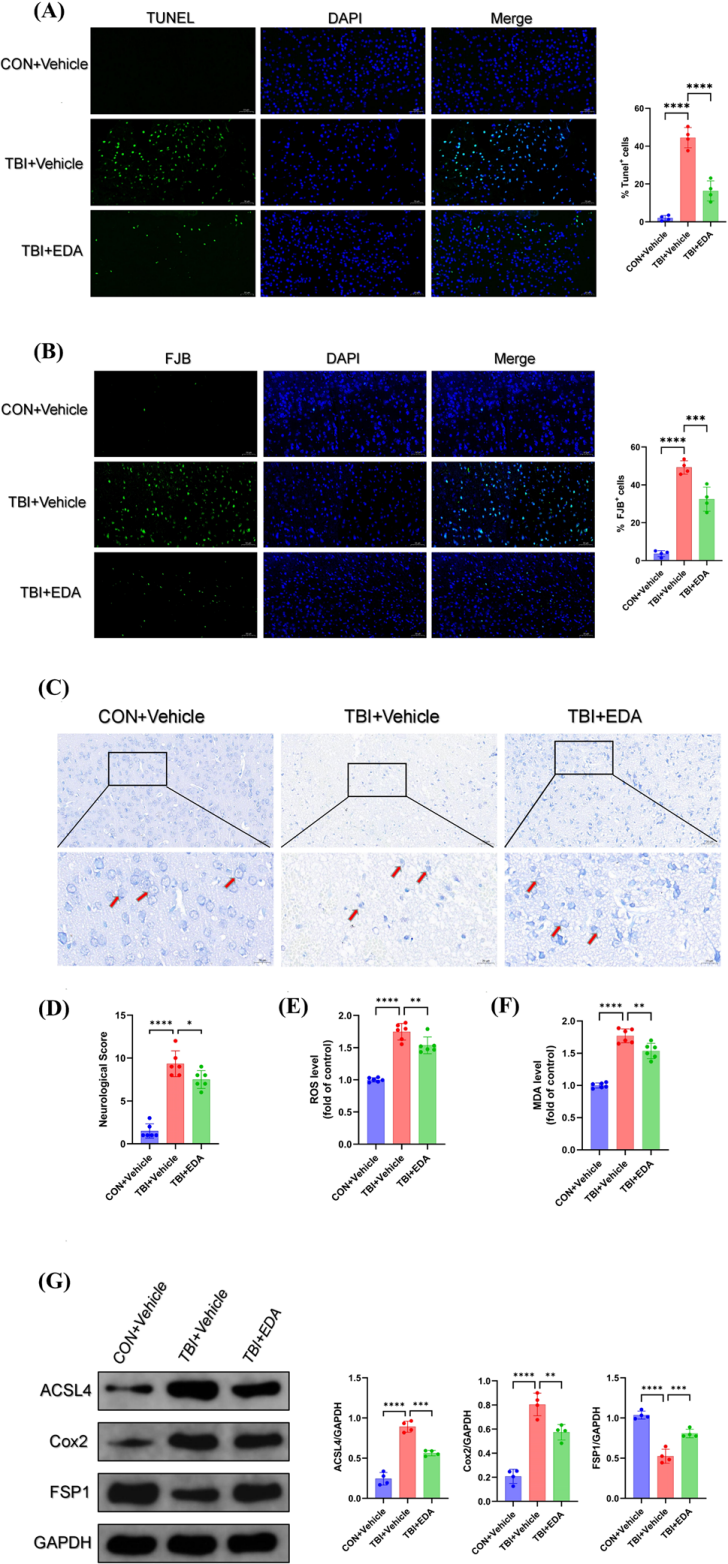
Edaravone alleviated neuronal damage and ferroptosis after TBI. **A** TUNEL staining (*n* = 4, scale bar 50 µm), **B** FJB staining (*n* = 4, scale bar 50 µm), and **C** Nissl staining of ipsilateral cortex of mice after TBI with treatment of edaravone (scale bar 20 µm). **D** mNSS scores reflecting behavioural changes of mice at TBI with treatment of edaravone (*n* = 6). **E** Lipid peroxidation indexes ROS (*n* = 6) and **F** MDA of mice after TBI with treatment of edaravone (*n* = 6). G Ferroptosis-associated protein detection of brain of mice after TBI with treatment of edaravone (*n* = 4). *, **, ***, and **** indicate *p* < 0.05, 0.01, 0.001, and 0.0001, respectively

To evaluate the therapeutic effects of EDA on early stage of TBI, we used TUNEL, FJB, and Nissl staining to observe pathological changes. After EDA administration, a reduction in neuronal apoptosis and degeneration (Figs. 3A and 2B) raised number of Nissl vesicles were observed after treatment of EDA (Fig. 3C). Furthermore, an improvement of mice behavioural scores was observed following EDA treatment (Fig. 3D), confirming capability of EDA to provide neuronal protection. Our analysis also revealed that EDA was capable of reducing neuronal lipid peroxidation and inhibiting neuronal ferroptosis by detecting lipid peroxidation levels of ROS, MDA (Fig. 3E,F), and ferroptosis-associated proteins ACSL4, Cox2, and FSP1 (Fig. 3G).

### Mechanical Injury Was One Cause of Neuronal Ferroptosis After TBI

**Fig. 4:**
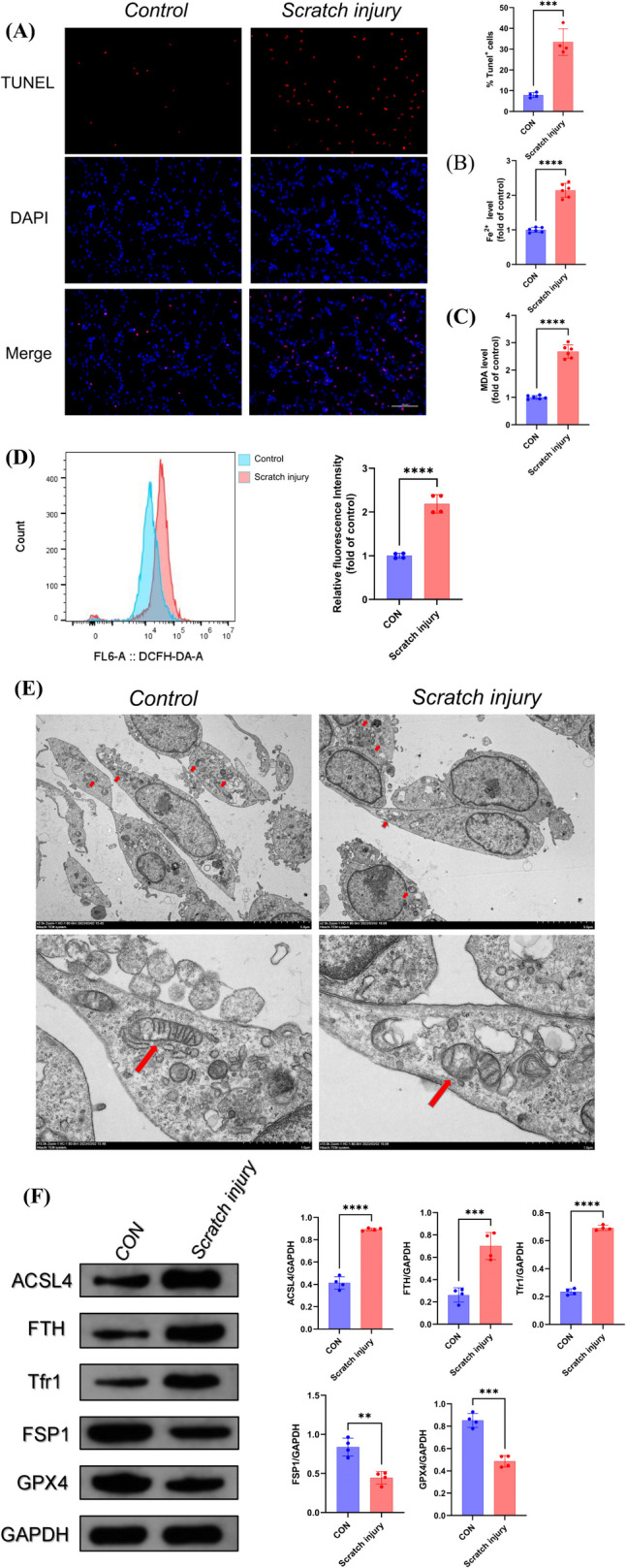
Mechanical injury contributed to neuronal ferroptosis after TBI. **A** TUNEL staining of PC12 cell line 12 h post scratching (*n* = 4, scale bar 100 µm). **B** Iron accumulation (*n* = 6), **C** lipid peroxidation indexes ROS (*n* = 6), and **D** MDA of PC12 cell line after scratching (*n* = 4). **E** transmission electron microscopy on scratched PC12 cells. **F** Ferroptosis-associated proteins detection on scratched PC12 cells (*n* = 4). *, **, ***, and **** indicate *p* < 0.05, 0.01, 0.001, and 0.0001, respectively

Since ferroptosis plays an important role in TBI, it is crucial to investigate the mechanism of ferroptosis occurring in injured neurons. We hypothesised that direct mechanical injury caused by TBI could be one of the factors inducing neuronal ferroptosis. The PC12 cell line was treated by scratching treatment and showed apoptosis at 12 h through TUNEL staining (Fig. 4A). Given that lipid peroxidation and iron metabolism disorders were two major biochemical features of ferroptosis, Fe^2+^ accumulation, ROS and MDA content were also detected. As expected, the content of Fe^2+^, ROS, and MDA was elevated with significantly raised after scratching injury (Fig. 4B–D). Moreover, we performed transmission electron microscopy on scratched PC12 cells and found ferroptosis mitochondria-specific changes, including rupture of outer membrane, volume reduction, mitochondrial cristae rupture, and increase in mitochondrial membrane density (Fig. 4E). At the same time, the ferroptosis-associated protein detection showed that ACSL4, FTH, and Tfr1 were elevated and GPX4 and FSP1 were reduced after onset of ferroptosis (Fig. 4F). This evidence proved that ferroptosis occurred after mechanical mechanistic injury caused by TBI.

### Edaravone Rescued Mechanical Scratching Induced Ferroptosis In Vitro

**Fig. 5:**
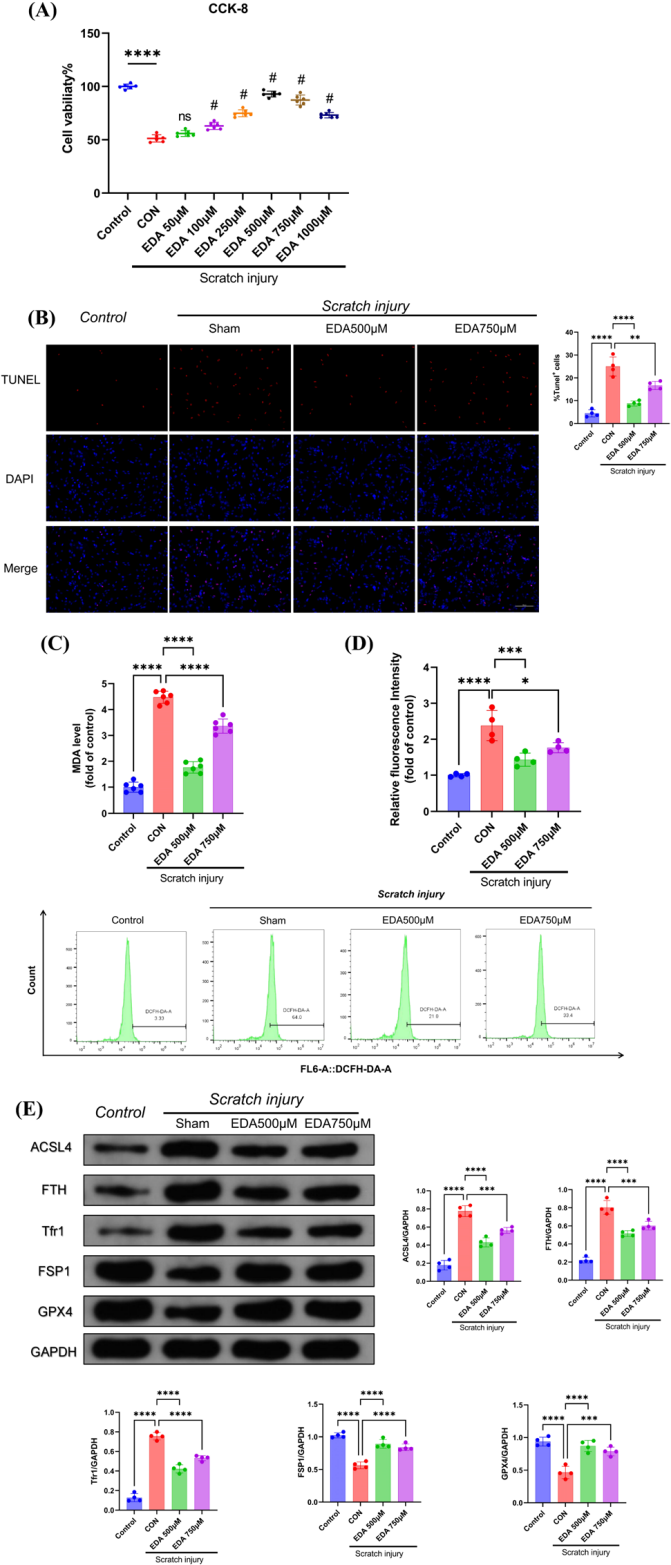
Edaravone alleviated mechanical scratching induced ferroptosis of PC12 cell line. **A** PC12 cell viability after scratching with 500 µM and 750 µM EDA intervention (*n* = 6). **B** TUNEL staining of PC12 cell line 12 h post scratching with 500 µM and 750 µM EDA intervention (*n* = 6, scale bar 100 µm). **C** Lipid peroxidation ROS (*n* = 6) and **D** MDA of PC12 cell line after scratching with 500 µM and 750 µM EDA (*n* = 4). **E** Ferroptosis-associated protein detection on scratched PC12 cells with 500 µM and 750 µM EDA intervention (*n* = 4). *, **, ***, and **** indicate *p* < 0.05, 0.01, 0.001, and 0.0001, respectively

In order to select the optimal dose of EDA, we used gradual content of EDA to treat PC12 cell line before scratching to assess the cell survival rate by CCK-8 method. The results showed cell viability was maximumly rescued when used 500 µM and 750 µM EDA intervention (Fig. 5A). Firstly, we observed the cell viability by TUNEL staining (Fig. 5B) and found that both 500 µM and 750 µM of EDA could reduce the number of TUNEL-positive cells. Subsequently, we are interested about whether EDA could inhibit ferroptosis in scratching model and found that not only the lipid peroxidation indicators ROS and MDA, but also ferroptosis-related proteins ACSL4, FTH, Tfr1, FSP1, and GPX4 could be reversed by EDA intervention after mechanical injury in vitro (Fig. 5C–E). These results suggest that EDA could inhibit ferroptosis and alleviate the adverse effects of PC12 cells after mechanical injury.

### Edaravone Rescued Mechanical Scratch-induced Ferroptosis by FSP1 Pathway In Vitro

**Fig. 6:**
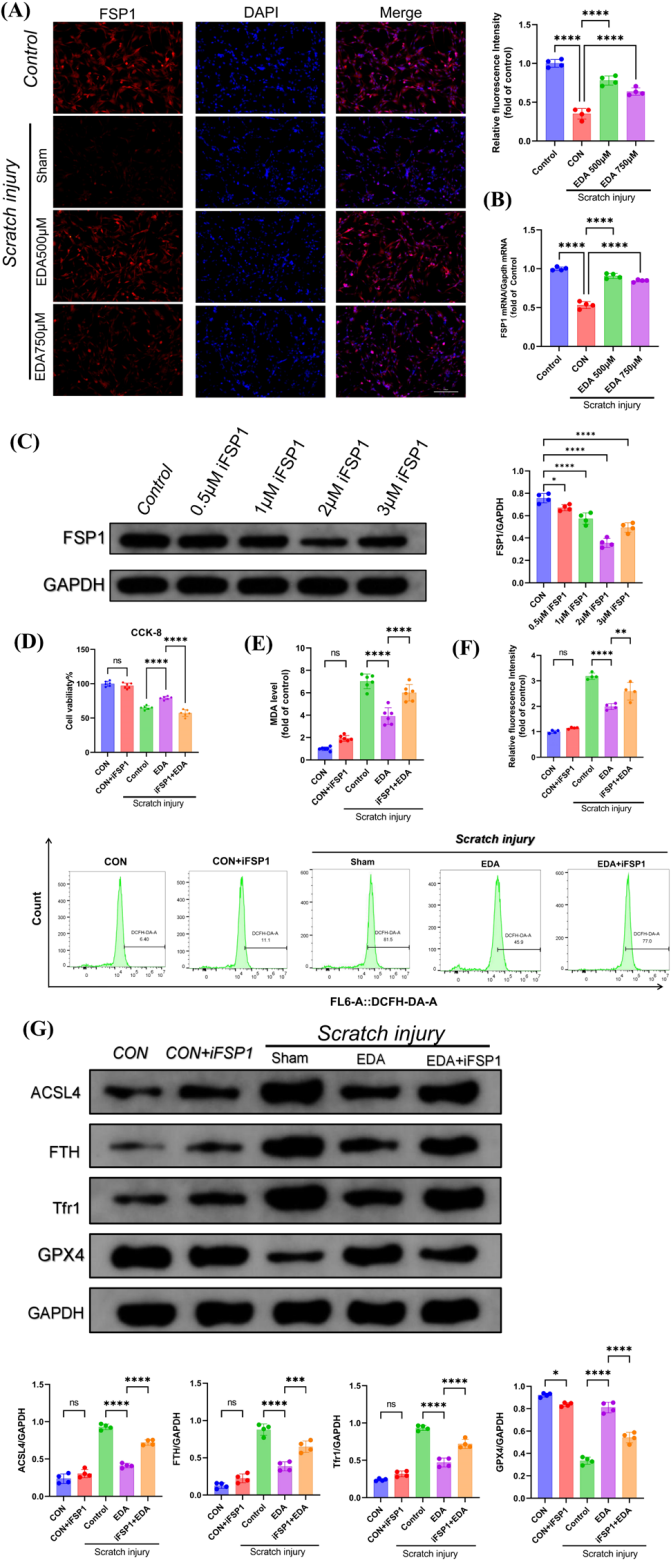
Edaravone rescued mechanical scratch-induced ferroptosis by FSP1 pathway of PC12 cells. **A** FSP1 protein immunofluorescence (*n* = 4, scale bar 100 µm) and **B** FSP1 mRNA levels of scratched PC12 cells with EDA (*n* = 4). **C** FSP1 protein levels of PC12 cells with iFSP1 agent (*n* = 4). **D** cell viability of PC12 cell line in scratching model with EDA treatment and iFSP1 intervention (*n* = 6). **E** Lipid peroxidation ROS (*n* = 6) and **F** MDA of PC12 cell line after scratching with EDA and iFSP1 intervention (*n* = 4). **G** Ferroptosis-associated protein detection on scratched PC12 cells with EDA and iFSP1 intervention (*n* = 4). *, **, ***, and **** indicate *p* < 0.05, 0.01, 0.001, and 0.0001, respectively

Since our previous study suggested that FSP1 could play a certain protective role in cerebral ischaemia–reperfusion injury [25], as well as FSP1 is an important regulatory protein for ferroptosis inhibition [37], we hypothesised that FSP1 mediated the treatment of EDA on TBI-induced ferroptosis. Our results revealed that FSP1 protein expression was altered using WB assays both in vitro and in vivo after EDA administration (Fig. 6G and E). By immunofluorescence and qRT-PCR detection, we prove that FSP1 protein was also significantly overexpressed after treatment with EDA (Fig. 6A and B), suggesting EDA probably inhibited neuronal ferroptosis via the FSP1 pathway. To verify this hypothesis, we transfected PC12 cell line with or without iFSP1, a new identified potent FSP1 inhibitor, and found that the concentration of iFSP1 at 2 µM reached the optimal effect on FSP1 inhibition (Fig. 6C). iFSP1 intervention also diminished therapeutic effect of EDA on cell viability of PC12 cell line in scratching model (Fig. 6D). As expected, lipid peroxidation metrics ROS, MDA, and ferroptosis biomarkers including ACSL4, FTH, Tfr1, and GPX4 in PC12 cells treated with EDA were reversed after FSP1 inhibition (Fig. 6E–G). In addition, the above indexes were not significantly altered after iFSP1 treatment of PC12 cells (Fig. 6D–G), inferring that iFSP1 had no adverse effect. These results suggested that EDA attenuated PC12 cells adverse effects of scratch-induced lipid peroxidation and ferroptosis, in which FSP1 mediated the EDA therapeutic effects.

## Discussion

Irreversible neuronal damage is a key factor contributing to neurological dysfunction as well as poor prognosis of TBI. In this study, we reported the presence of neuronal ferroptosis in the early stages of TBI and demonstrated the early protective effect of EDA against TBI. In addition, we preliminarily elucidated the causal relationship between mechanical stress injury and early neuronal ferroptosis in TBI in vivo an in vitro and demonstrated that EDA inhibited the occurrence of neuronal ferroptosis at least through the FSP1 pathway.

Edaravone (EDA), as a neuroprotective drug, has been evaluated in studies for its therapeutic effects on chronic degenerative diseases such as stroke, Alzheimer’s disease, vascular dementia, and depression. Studies on its protective mechanisms have focused on its resistance to oxidative stress and anti-inflammatory effects [18, 38]. However, few studies have focused on the therapeutic effects of EDA on TBI and the neuroprotective effects targeting ferroptosis. In our study, pathological changes of cortical neurons in the damaged region and accumulation of ions in brain tissue were observed on the 1st, 3rd, and 5th day of acute phase of TBI. The ferroptosis-related protein was also present in the acute phase of TBI. Notably, in the absence of therapeutic interventions TBI mice did not repair damage simultaneously. Based on previous studies on EDA drug half-life [19, 30], we focused on the therapeutic effect of EDA in the acute phase of TBI, of which animals received EDA (10 mg/kg intraperitoneally) 24 h and 30 min before, 2 h, 12 h, 24 h, 48 h, and 72 h after TBI. It was shown that administration of EDA significantly attenuated TBI-induced ferroptosis and reversed a series of cortical histopathological changes and behavioural deficits. Future studies will focus on determining the time course and duration of ferroptosis-related protein expression after TBI, as well as investigating the effects of prolonged EDA treatment (such as 14 days, 21 days) to improve secondary disorders, such as cognitive deficits and secondary epilepsy, associated with TBI. Whatever, we found EDA, which is mainly used in the prognostic treatment of hemorrhagic stroke, perform neuroprotective via inhibition of ferroptosis after TBI in this study.

Lipid peroxidation, one of the typical features of ferroptosis, is an important indicator for assessing EDA drug efficacy. The level of lipid peroxidation can be assessed mainly by measuring its metabolites like ROS and MDA, but also anti-lipid peroxides, and studies have shown that GPX4 and FSP1, the main regulatory factors of anti-lipid peroxidation in ferroptosis [39, 40], and ACSL4, a key enzyme in the lipid peroxidation pathway [41, 42], reflect the level of ferroptosis. Meanwhile, it is also an important indicator of lipid peroxidation levels.

Disorders of iron metabolism is another typical feature of ferroptosis, and monitoring of iron metabolism changes is also a supplementary detection for the ferroptosis and pharmacological efficacy after TBI. Iron absorption, transport, and storage are closely related to the maintenance of iron homeostasis, including transferrin receptor (Tfr1) and ferritin heavy chain (FTH). In previous studies, FTH was suggested to store excess iron and have some iron oxidase activity to stabilise free divalent iron, leading to protection for cells from abnormal iron metabolism damage [33]. Interestingly, a clinical study found that FTH expression levels were strongly correlated with GCS scores and mortality in TBI [11]. Meanwhile, FTH can be delivered to lysosomes by target binding to NCOA4 for degradation and release free unstable divalent iron, increasing the possibility of ferroautophagy-induced ferroptosis [43]. In present study, we found expression level of FTH increased significantly after TBI in vivo and in vitro, mainly because of the perspective of iron overload after TBI, which is a potential research worth focus. PTGS2, also known as COX2, is an important marker for evaluating ferroptosis and is a key enzyme in the prostaglandin biosynthesis pathway, which also acts as a peroxidase and dioxygenase in biometabolic pathways [44]. In this experiment, we monitored ferroptosis by targeting the lipid peroxidation indicators ROS, MDA, FSP1, GPX4, and ACSL4 and the iron metabolism indicators Tfr1 and FTH, and further COX2 expression. All the biomarkers showed ferroptosis occurred after TBI in vitro and in vivo.

The relationship between the pathological course of TBI and neuronal ferroptosis has been widely reported, and inhibition of neuronal ferroptosis is strongly associated with the prognosis of TBI [9, 45]. However, studies on the mechanism of neuronal ferroptosis after TBI need to be explored. Some studies have suggested that haemoglobin accumulates in tissues after TBI injury due to the rupture of intracranial microvessels induced by TBI, and haemoglobin releases a large amount of free iron under haemoglobin oxygenase [12, 14, 46, 47], in which process free irons can be phagocytosed by microglial cells and lead to ferroptosis [35] and ultimately a series of cascade responses. Although disturbed homeostasis and a series of cascade reactions are the main causes of neuronal ferroptosis after TBI, this mechanism about haemoglobin accumulates is similar to that after haemorrhagic stroke, which does not virtually reflect the nature of TBI injury. Therefore, in this experiment, we established a cell scratching model to investigate whether the mechanical injury in TBI could also lead to the occurrence of ferroptosis. After mechanical scratching, both the level of iron metabolism disorders and lipid peroxidation levels occurred in line with the trend of ferroptosis, and we observed the mitochondrial morphology was consistent with the morphological changes induced by ferroptosis [48], preliminarily providing evidence that direct mechanical injury from TBI also contributes to neuronal ferroptosis. Moreover, we performed EDA therapeutic intervention and also observed that EDA had an effect on cell viability, pathological changes, and neural ferroptosis after scratching.

In our previous study, we have observed the cellular localization of FSP1, which is mainly localised to the cell membrane, and is also present in other organelles of cytoplasm such as mitochondria, endoplasmic reticulum, and Golgi apparatus. And a link between the process of cellular iron death and altered levels of FSP1 expression has also been observed [25]. Doll et al. [23] identified a protein in GSH-independent pathway regulating ferroptosis, naming it ferroptosis suppressor protein 1 (FSP1), which plays an indispensable role in scavenging lipid peroxidation after ferroptosis and is the second component in ferroptosis inhibition after XC-system/GSH/GPX4 pathway. Its main mechanism is to exert its NAD(P)H oxidase to convert ubiquinone (CoQ10) in the cell membrane to reduced ubiquinol, thus trapping oxygen radicals to antagonise lipid peroxidation and ultimately play a protective role during cellular ferroptosis [40]. Meanwhile, FSP1 may also inhibit ferroptosis through the Vk cycle pathway [49] or participate in the repair of the ESCRT-III-mediated membrane repair pathway after ferroptosis [50, 51]. To address the protective mechanism of FSP1 against cellular ferroptosis, more research work is needed to do in the future. Based on the antagonistic effect of FSP1 against ferroptosis, we previously proved the protective effect of FSP1 after neuronal injury [25, 37]. Also, EDA can antagonise oxygen radicals via the Sirt1-Nrf2 pathway [19], in which two antioxidant response elements (AREs) have been identified upstream of the FSP1 transcriptional initial site, validating of the association of Nrf2 and FSP1 [24]. We found that FSP1 changed significantly after EDA treatment of TBI in both cellular and animal models. Therefore, we speculated that the mechanism of action of EDA may be to activate the FSP1 pathway, to inhibit the occurrence of neuronal ferroptosis, then to achieve the therapeutic effect. In the present study, we only preliminarily elucidated that the mechanism of action of EDA is to inhibit the occurrence of neuronal ferroptosis due to TBI mechanical injury partly through the FSP1 pathway to achieve therapeutic effect. However, the complex intermediate molecular pathways need to be explored in subsequent studies. In the follow-up experiments, we propose the hypothesis that EDA inhibits neuronal iron death via the Sirt1-Nrf2-FSP1 pathway and will conduct further in-depth studies along this trail.

**Fig. 7:**
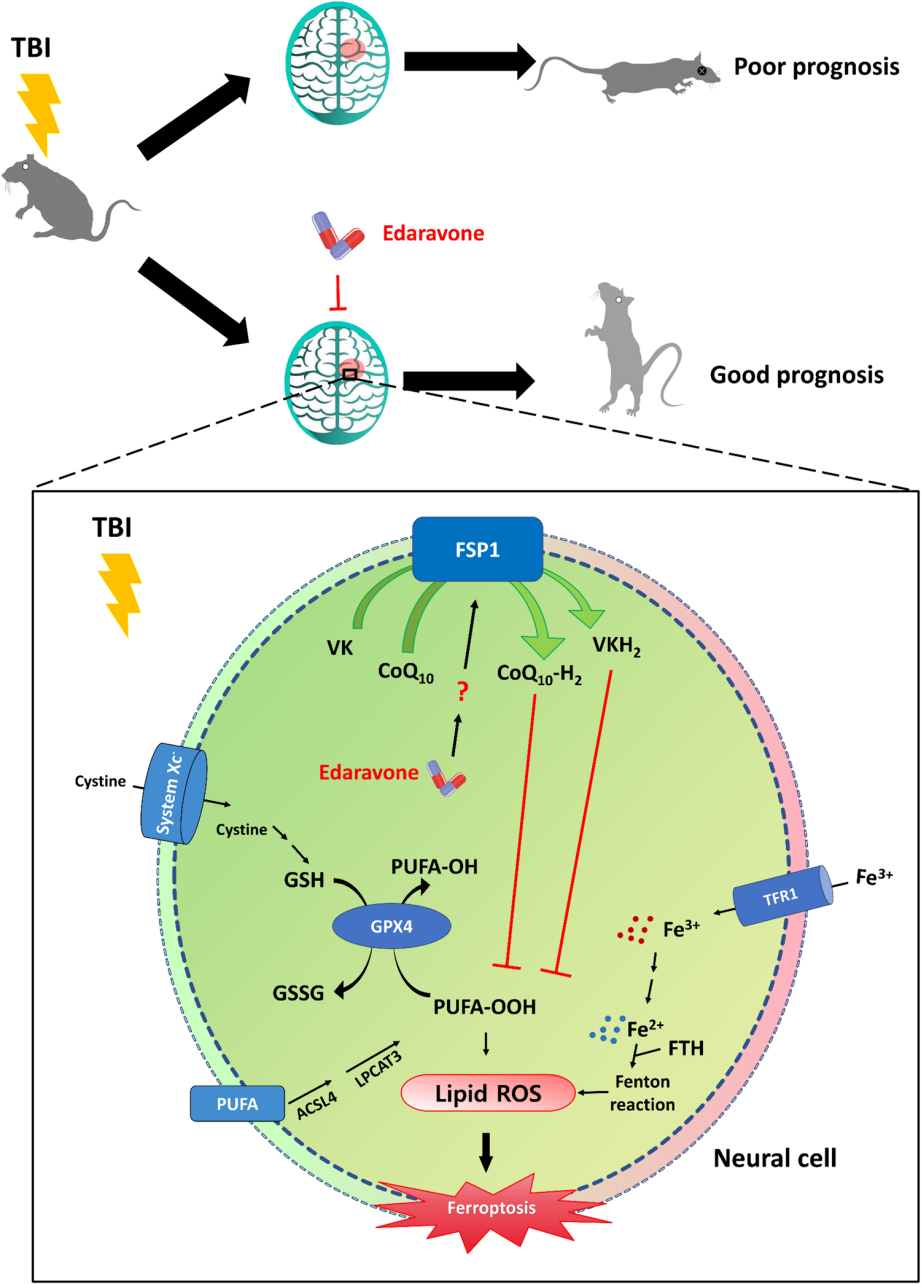
Schematic illustration of the mechanisms underlying EDA-related anti-ferroptosis efficacy. During the process of ferroptosis, polyunsaturated fatty acids (PUFA) was changed to ROS by several biochemical steps, while Fe^2+^ also produces ROS through the Fenton reaction, which further exacerbates lipid peroxidation reaction [7]. Meanwhile, two major pathways regulating ferroptosis, including the XC-system/GSH/GPX4 axis synthesizing and utilizing GSH [39] and FSP1 on membrane generating CoQ_10_-H_2_, VK-H_2_, etc., inhibits lipid peroxidation [23, 40, 49]. In TBI-induced ferroptosis, EDA activates FSP1 in unknown mechanism, thereby enhancing the inhibitory effect on lipid peroxidation and ferroptosis, and thus attenuating neuronal damage

In this study, we demonstrated the therapeutic properties of EDA against neuronal ferroptosis in TBI and its underlying mechanisms (Fig. 7). In order to investigate whether EDA achieves cerebroprotective effects by targeting FSP1 pathway, we used iFSP1, a newly discovered ferroptosis agonist which mainly competitively inhibits the upstream of degrading enzyme of FSP1, maintaining degrading enzyme of FSP1 activity, to achieve the effect of FSP1 inhibition [23]. In our experiments, we pretreated PC12 cells with iFSP1 to interfere with FSP1 expression. Interestingly, we found that the inhibitory effect of iFSP1 on FSP1 was not concentration-dependent, which is in line with the off-target effect when the concentration of iFSP1 is higher as found by Nakamura et al. [52]. Moreover, we found that the mechanical scratching of PC12 cells with iFSP1 pretreatment failed to reverse the therapeutic effects by EDA, which preliminarily elucidated that EDA could inhibit the occurrence of neuronal ferroptosis at least through the FSP1 pathway. However, no reports revealed the effect of iFSP1 on neuronal cell function. In this experiment, we found that interfering with PC12 cells by adding appropriate amounts of iFSP1 did not affect cell viability as well as lipid peroxidation. In future studies, we expect to use icFSP1, an emerging FSP1 inhibitor which performed good effects in vivo by triggering the phase separation of FSP1 and inhibited FSP1 function [52], to explore the efficiency of icFSP1 and protective significance of FSP1 in TBI experiments.

## Conclusion

In conclusion, there are various mechanisms for the occurrence of neuronal ferroptosis after TBI, and we found that the direct mechanical injury may be the initiating factor of neuronal ferroptosis in the early stage of TBI. Meanwhile, EDA, as a multifunctional cerebroprotective agent, could exert its neuroprotective effects through multiple protective mechanisms in the treatment of TBI. In this experiment, we not only provided evidence that EDA produced cerebroprotective effects in the acute phase of TBI, but also demonstrate that EDA could inhibit ferroptosis by acting on the FSP1 pathway in neurons.

## Data Availability

The datasets generated and analysed during the current study are available upon reasonable request.
